# Development and validation of the physical fitness test anxiety scale for college students

**DOI:** 10.3389/fpsyg.2025.1573530

**Published:** 2025-04-08

**Authors:** Shanshan Yin, Fang Tang, Pincao Gao

**Affiliations:** ^1^Ministry of Public Sports, Taizhou University, Taizhou, China; ^2^College of Physical Education, Anqing Normal University, Anqing, China

**Keywords:** college students, physical fitness test anxiety, scale development, reliability and validity testing, factor analysis

## Abstract

**Objective:**

This study aims to develop and validate a Physical Fitness Test Anxiety Scale (FTAS) for Chinese college students and assess its scientificity and applicability through reliability and validity testing.

**Methods:**

The initial scale was constructed through literature analysis and semi-structured interviews, followed by item analysis and factor analysis to optimize the scale structure. Multiple statistical methods were used to test the scale’s reliability and validity.

**Results:**

The final scale consists of 12 items, covering two dimensions: cognitive anxiety and somatic anxiety. The model fit was good (*χ*^2^/df = 2.29, RMSEA = 0.051, IFI, CFI, TLI, GFI, AGFI >0.90). The internal consistency coefficients for the total scale and the two dimensions ranged from 0.903 to 0.928, with split-half reliability between 0.804 and 0.899. The average variance extracted (AVE) ranged from 0.575 to 0.667, and the composite reliability (CR) ranged from 0.904 to 0.950.

**Conclusion:**

The Physical Fitness Test Anxiety Scale for college students has good reliability and validity and is suitable for assessing the physical fitness test anxiety levels of college students.

## 1 Introduction

In recent years, standardized physical fitness tests have been widely adopted as a crucial tool for assessing students’ physical health within educational systems globally (Till et al., 2023; Kljajević et al., 2021). Initiatives such as the FITNESSGRAM® in the United States, the ALPHA project in the European Union, and physical fitness testing systems across various Asian countries underscore the high priority placed on monitoring the physical health of adolescents (Lee et al., 2020; Yan et al., 2020; Fühner et al., 2021). However, these physical fitness tests may not fully achieve their intended purpose of promoting physical and mental health. Instead, their exam-oriented nature can exacerbate students’ psychological stress and anxiety levels (Sabe et al., 2022; Huhtiniemi et al., 2021; Huhtiniemi et al., 2022). Research indicates that anxiety induced by physical fitness tests can lead to test avoidance and negative coping behaviors, and may also cause lasting harm to mental health through chronic stress mechanisms (Simonton et al., 2019; Asif et al., 2020). The negative impact of such psychological stress responses often outweighs the physical fitness deficits themselves, creating a dual health risk of “psychological-physiological” nature—when students avoid physical activity due to anxiety, they not only miss opportunities to improve their physical fitness but may also fall into a vicious cycle of “anxiety-avoidance-declining fitness-increasing anxiety” (McDowell et al., 2019). Therefore, the anxiety triggered by physical fitness tests and its underlying psychological mechanisms have become an important topic requiring in-depth research in the field of sports psychology.

Although the issue of fitness test anxiety has gained increasing attention in recent years, existing research primarily focuses on athletes’ competition-related anxiety or physical fitness test anxiety in adolescents (Li et al., 2020; Carter et al., 2021; Kaplánová, 2021). These studies often center on athletes’ competitive performance or the anxiety responses of adolescents in fitness testing scenarios. However, mental health issues among students, particularly anxiety symptoms among university students, have become a significant concern in global mental health (Li et al., 2022; Dessauvagie et al., 2022).As a distinct social group, university students face multiple challenges including academic pressure, physical health assessments, and societal expectations, making their mental health a critical area of concern (Sheldon et al., 2021; Hernández-Torrano et al., 2020). In this context, research on university students’ anxiety experiences during physical fitness tests, particularly their unique psychological mechanisms and influencing factors, remains scarce.

At present, there are several mature tools available for measuring anxiety in sports contexts, such as the State–Trait Anxiety Inventory (STAI), Sport Anxiety Scale (SAS), Sports Competition Anxiety Test (SCAT), and Competitive State Anxiety Inventory-2 (CSAI-2) (Spielberger et al., 1970; Smith et al., 1990; Martens, 1977; Martens et al., 1990).However, these scales have several limitations: First, existing scales are primarily designed for competitive sports contexts, with measurement dimensions and indicators mainly reflecting athletes’ anxiety states during competitions, thus failing to capture the unique aspects of university students’ physical fitness testing. Second, these scales do not adequately consider the distinctive features of physical fitness tests as standardized, periodic assessments closely linked to academic evaluations (Yin et al., 2022). Third, compared to competitive anxiety, university students’ anxiety during physical fitness tests is influenced by more complex factors, including test requirements, grading standards, and self-perceptions of physical performance (Huhtiniemi et al., 2021; Yin et al., 2022).

From a psychological mechanism perspective, anxiety during physical fitness tests may involve multiple psychological processes, including cognitive appraisal, emotional experience, physiological responses, and behavioral manifestations (Roos et al., 2023; Lin and Gao, 2023). While existing scales touch on these aspects to some extent, they lack a systematic theoretical framework to explain the interconnections among these psychological mechanisms and their comprehensive impact on physical fitness test anxiety. A deeper understanding of these underlying psychological mechanisms is essential not only for the theoretical construction of scale development but also for providing clearer analytical directions for subsequent research.

Therefore, existing scales are inadequate for accurately capturing university students’ anxiety experiences during physical fitness tests and cannot fully reveal their psychological characteristics and mechanisms. Developing a measurement tool specifically tailored for university students, which thoroughly considers the unique aspects of physical fitness testing, holds significant theoretical and practical value. Specifically, the study will conduct a systematic review of the relevant literature and conduct interviews with college students to explore the connotations and structural dimensions of fitness test anxiety. Following the established guidelines for scale development, we will design and validate an assessment tool that is culturally and educationally appropriate for China. The results of this study will not only contribute to a deeper understanding of the psychological mechanisms of college students’ fitness test anxiety, but also provide theoretical and practical guidance for optimizing fitness test evaluation systems in higher education and developing effective anxiety intervention strategies. Ultimately, these efforts will contribute to better supporting the physical and mental well-being of college students.

## 2 Conceptualization and dimensions

### 2.1 Definition of physical fitness test anxiety

Physical fitness test anxiety is an important construct in the field of sports psychology, and its conceptual definition requires a systematic review from a theoretical perspective, as well as a clear distinction from related concepts. Based on a comprehensive review of existing literature and the specific context of higher education in China, this study provides a theoretical definition of physical fitness test anxiety.

Firstly, physical fitness test anxiety differs fundamentally from sport anxiety. Sport anxiety primarily arises from concerns about performance and skill evaluation in competitive sports, with the core characteristic being the tension and uncertainty associated with competition contexts (Smith et al., 1990; Kaplánová, 2021). In contrast, physical fitness test anxiety refers to specific anxiety responses experienced by individuals when confronted with standardized physical fitness tests. This anxiety primarily stems from concerns and expectations regarding the test results (Huhtiniemi et al., 2021; Yin et al., 2022). Furthermore, physical fitness test anxiety is distinctly different from sports exam anxiety. Sports exam anxiety encompasses a broader range of concerns, including evaluation of both athletic skills and physical performance, as well as overall performance assessment in physical education courses (Danthony et al., 2020; Mascret et al., 2021). In contrast, physical fitness test anxiety is more narrowly focused on standardized testing situations, where the core concern is the achievement of specific test standards and the potential consequences of the test results (Yan et al., 2020; Simonton et al., 2019).

In the context of Chinese higher education, the National Student Physical Fitness Standard (NSPFS) provides an essential situational framework for understanding physical fitness test anxiety (Yan et al., 2020). This standardized system assesses students’ physical fitness through various indicators, including the standing long jump (strength), 50-meter sprint (speed), 800/1000-meter run (endurance), and sit-and-reach (flexibility), along with health metrics such as body mass index (BMI) and lung capacity. The NSPFS results not only determine whether students meet physical health standards but also have direct implications for academic evaluations such as awards, honors, and graduation eligibility, thereby intensifying students’ anxiety (Yin et al., 2022).Therefore, based on the multidimensional theory of anxiety (Spielberger et al., 1983), this study defines physical fitness test anxiety as: the negative psychological states of tension, worry, and unease that students experience in anticipation of and in response to the process, results, and consequences of the NSPFS tests.

### 2.2 Structure dimensions of physical fitness test anxiety

This study systematically explored the structural dimensions of physical fitness test anxiety among university students through a review of the literature and semi-structured interviews. The research was divided into three stages: first, a systematic analysis of existing sports anxiety scales to identify common structures of anxiety in sports contexts; second, interviews to gather primary data and gain deeper insights into students’ experiences of anxiety during physical fitness tests; and third, the integration of findings from the literature and interviews to propose a theoretical framework of university students’ physical fitness test anxiety, providing a foundation for subsequent scale development.

#### 2.2.1 Review of relevant sports anxiety scales

To construct a theoretical framework for physical fitness test anxiety, the study first conducted a systematic review of existing sports anxiety scales. Using keywords such as “sports anxiety scale,” “sports anxiety measurement,” “college students’ sports anxiety,” and “sports anxiety review,” relevant literature was retrieved from databases such as Web of Science, PsycINFO, SPORTDiscus, and CNKI (China National Knowledge Infrastructure). From an initial pool of 183 articles, three rounds of screening excluded irrelevant studies, non-sports anxiety research, and studies that did not use Likert-type scales. Finally, 12 high-quality articles were selected for analysis.

Then, an expert panel comprising one senior psychology professor and two doctoral students conducted an in-depth analysis of these scales. The doctoral students independently summarized the scale items and extracted key dimensions of sports-related anxiety, while the professor moderated a discussion to evaluate the rationality and consistency of the categorizations and resolved any disagreements. The analysis revealed that traditional sports anxiety research predominantly focuses on two dimensions: cognitive anxiety and somatic anxiety (Smith et al., 1990; Martens, 1977). Cognitive anxiety refers to an individual’s negative expectations, self-doubt, and fear of failure, which affect their psychological state and decision-making. Somatic anxiety, on the other hand, refers to physiological tension and symptoms caused by autonomic nervous system arousal, such as increased heart rate, sweating, and muscle tension during or before the test. Based on these findings, this study constructed a framework for measuring physical fitness test anxiety using these two dimensions. This framework provides a theoretical basis for further exploration of the structural dimensions of physical fitness test anxiety and enhances our understanding of the construct.

#### 2.2.2 Field interviews with university students

Based on the two-dimensional model of cognitive anxiety and somatic anxiety established through the literature review, this study further focused on the specific context of fitness testing to explore the model’s applicability. A stratified random sampling method was employed to select 10 university students from three major regions in China (East China, South China, and Northwest China), ensuring diversity and representativeness in terms of gender balance and academic disciplines (including humanities, sciences, and engineering). Semi-structured interviews were conducted, each lasting approximately 60 min, with the interview guidelines reviewed and refined by an expert panel. The interviews centered on the dimensions of cognitive and somatic anxiety. Cognitive Anxiety: Questions focused on whether students experienced anxiety during fitness testing, whether they were concerned about poor performance affecting academic evaluations, and whether they encountered cognitive burdens such as difficulty concentrating. Somatic Anxiety: Students were asked whether they exhibited physiological responses during fitness testing, such as increased heart rate, sweating, rapid breathing, or muscle tension.

The results revealed that cognitive anxiety was prevalent among students. All participants expressed varying degrees of concern about their fitness test results, with 70% (7/10) worrying primarily about their own performance, 20% (2/10) specifically concerned about test scores affecting eligibility for awards, and 10% (1/10) final-year students fearing that failing the test might jeopardize graduation. Somatic anxiety was also prominent, with 80% (8/10) of students reporting at least one physiological anxiety symptom. Specifically, 50% (5/10) experienced trembling hands or feet, 70% (7/10) reported increased heart rate, and 20% (2/10) experienced nausea or vomiting after long-distance running tests. These findings not only validate the applicability of the traditional two-dimensional anxiety model to the context of fitness testing but also provide critical insights for refining the scale in subsequent stages. Table 1 summarizes key excerpts from the interviews.

**Table 1:** Basic information of university students and selected interview content.

Subject	Region	Gender	Faculty	Dimension	Raw data and summary
1	Jiangsu	Female	School of education sciences	Somatic anxiety Cognitive anxiety	“I hardly eat for an entire week before the fitness test because I lose my appetite, especially before the 800-meter run. The last time I ran 800 meters, I coughed for half a month afterward—it was so painful. My roommate even suspected that I might have injured my lungs.”
2	Anhui	Male	School of mathematics and science	Cognitive anxiety	“I feel extremely anxious before the fitness test and imagine all sorts of bad scenarios related to it. During the test, I worry about my performance and feel quite anxious, but once it’s over, I feel more relaxed.”
3	Hunan	Female	School of humanities	Cognitive anxiety Somatic anxiety	“I think the anxiety grows exponentially when fitness tests are tied to grades or graduation requirements. Whenever there’s a long-distance run, my anxiety doubles. If there’s no long-distance running, it’s manageable, but with it, I feel like I’m doomed.”
4	Guangxi	Female	School of economics and management	Cognitive anxiety	“When I feel anxious, I find myself worrying even more, even about things that I would not usually be concerned about. My thoughts also become more negative. For example, before a long-distance run, I might worry that my shoelaces will suddenly come undone during the run, or I might wonder if I’ll suddenly pull a muscle during the standing long jump.”
5	Yunnan	Female	School of music	Cognitive anxiety Somatic anxiety	“I really want to avoid the fitness test altogether. As soon as I think about the upcoming test, I lose interest in doing anything else. But when I finally step onto the field, my body feels so heavy, and running becomes so exhausting. This anxiety even affects my sleep. When I think about these things before bed, I feel utterly drained, extremely anxious, and so tired of life that I cannot sleep at all and end up staying awake all night.”

#### 2.2.3 The two-dimensional structure of fitness testing anxiety

Integrating findings from the literature review and interview study, this research adopts a two-dimensional structure of cognitive anxiety and somatic anxiety to explain the phenomenon of fitness testing anxiety. Cognitive anxiety primarily manifests as students’ concerns about their fitness test performance, aligning with the “performance-related anxiety” concept in test anxiety theories (Danthony et al., 2020). Somatic anxiety is characterized by physiological symptoms during the testing process, such as muscle tension and increased heart rate, consistent with previous research on physiological responses (Huhtiniemi et al., 2021; Yin et al., 2022).

Therefore, the scale development in this study focuses on these two dimensions, which clarifies the conceptualization of fitness testing anxiety and provides a solid theoretical foundation for the development of measurement tools and subsequent empirical studies.

## 3 Methods

### 3.1 Research design and participants

This study employed a cross-sectional design aimed at developing and validating a physical fitness test anxiety scale tailored for Chinese university students. The target population consisted of undergraduate students, and data collection was conducted using a stratified sampling method across six universities in three prefecture-level cities (Nanjing, Lianyungang, and Taizhou) in Jiangsu Province. The sampling procedure was as follows: First, universities were categorized into large (enrollment >20,000 students), medium (10000–20,000 students), and small (<10,000 students) based on their size, with two universities randomly selected from each category. Second, within each university, stratification was performed according to the nature of academic departments (science and engineering, humanities and social sciences, medical sciences, and arts and sports) to ensure representation across different academic disciplines. Finally, quota sampling was applied based on academic year (freshman to senior).

Questionnaire Distribution and Data Collection: the questionnaire was distributed through an online platform from October 19 to October 24, 2023. A total of 1,022 questionnaires were distributed, and 996 valid responses were collected, yielding a valid response rate of 97.46%. Invalid questionnaires were excluded based on the following criteria: (1) response time too short (less than 2 min) or too long (more than 30 min); (2) incomplete responses (missing values exceeding 10%); (3) abnormal response patterns (e.g., selecting the same option for all items); and (4) incorrect responses to reverse-coded items: the questionnaire included reverse-coded items to detect response validity, and responses that contradicted the logic of the positively worded items were deemed invalid. These criteria ensured the reliability and validity of the data.

The sample composition was as follows: in terms of gender, 486 participants (48.80%) were male, and 510 (51.20%) were female; regarding academic year, 268 (26.91%) were freshmen, 276 (27.71%) were sophomores, 242 (24.30%) were juniors, and 210 (21.08%) were seniors; by academic discipline, 412 (41.37%) were from science and engineering, 286 (28.71%) from humanities and social sciences, 168 (16.87%) from medical sciences, and 130 (13.05%) from arts and sports. In terms of physical activity participation, 42.57% engaged in exercise three or more times per week, 38.96% exercised one to two times per week, and 18.47% rarely exercised.

Inclusion criteria for participants were as follows: aged between 18 and 25 years, enrolled as full-time undergraduate students, having participated in the university’s physical fitness test, and voluntarily agreeing to participate in the study. Participants were also required to have no physiological or psychological disorders and be able to independently complete the questionnaire. Exclusion criteria included individuals with cognitive impairments or mental health conditions, and those unable to complete the physical fitness test.

### 3.2 Development process

Based on existing sports anxiety measurement tools, this study systematically conducted cross-contextual adaptation and localization of initial items to align with the standardized fitness testing context in Chinese universities. The specific process is as follows.

#### 3.2.1 Sources of scale items and contextual adaptation

Based on classical sports anxiety scales, this study selected items with cross-contextual applicability for adaptation. Key reference tools included the State–Trait Anxiety Inventory (STAI), Sport Anxiety Scale (SAS), Sports Competition Anxiety Test (SCAT), and Competitive State Anxiety Inventory-2 (CSAI-2) (Spielberger et al., 1970; Smith et al., 1990; Martens, 1977; Martens et al., 1990). The contextual adaptation involved three stages: first, the wording of items related to “competition scenarios,” “sports performance,” and “competitive pressure” was revised to better fit the fitness testing environment. For example, “I am worried about failing in the competition” was rephrased as “I am worried about performing poorly in the fitness test.” Second, the content of the scale was embedded within the unique NSPFS testing context in Chinese universities. For instance, “sports performance” was specified as “800-meter running performance” or “pull-up performance.” Finally, additional items reflecting institutional pressures were included, such as “Failing the test may affect academic evaluations.”

#### 3.2.2 Expert revision and content validity assessment

To ensure the scale’s professionalism and applicability, an expert panel consisting of one professor of sports psychology and two doctoral students with experience in scale development was formed. Using a double-blind cross-revision method, the panel refined the items with a focus on: 1. Semantic Adaptation: Adjusting terms like “competition outcomes” to “testing benchmarks.” 2. Cultural Appropriateness: Removing items related to coping strategies. 3. Dimensional Differentiation: Avoiding overlap between cognitive and somatic dimensions. Through three rounds of Delphi method evaluation, the panel deleted four semantically redundant items and merged three similarly phrased items, resulting in a 36-item preliminary scale encompassing two dimensions: Cognitive Anxiety, e.g., “I am worried about failing the fitness test.” Somatic Anxiety, e.g., “I experience stomach cramps before the test.”

#### 3.2.3 Pilot testing and item optimization

A stratified sample of 15 university students with NSPFS testing experience was recruited for cognitive interviews using the think-aloud method to examine item clarity. Based on the feedback:

1. Technical terms were simplified, e.g., “anticipatory anxiety” was changed to “nervousness before the test.” 2. Contextual prompts were added, e.g., clarifying that “test” refers to the NSPFS annual test. 3. Likert scale anchors were adjusted, replacing “strongly disagree - strongly agree” with “never - always” to enhance behavioral anchoring. The final version of the scale adopted a 5-point Likert format (1 = never, 5 = always) and consisted of 25 items, with each item closely aligned to its respective dimension, accurately measuring university students’ fitness testing anxiety.

### 3.3 Statistical methods

Data processing and analysis were conducted using SPSS 26.0 and AMOS 26.0. To ensure the independence and reliability of the data, the valid questionnaires (*n* = 996) were randomly divided into two equal subsamples: Subsample 1 (*n* = 498) was used for exploratory factor analysis (EFA), and Subsample 2 (*n* = 498) was used for confirmatory factor analysis (CFA). Preliminary tests indicated no significant differences between the two subsamples in terms of demographic variables, ensuring the validity of subsequent analyses. The structure of the Physical Fitness Test Anxiety Scale was evaluated through item analysis and exploratory factor analysis, and its robustness was further examined using confirmatory factor analysis. Additionally, internal consistency, split-half reliability, convergent validity, discriminant validity, and criterion-related validity were calculated to comprehensively assess the scale’s reliability and validity.

## 4 Results

### 4.1 Item analysis

In the item analysis phase, this study first summed up 25 initial items, the total score was divided into high group (top 27%) and low group (bottom 27%) by critical ratio method to test the discrimination degree of each item. The results of the independent sample t-test indicated that all items had a significance level of less than 0.001, and the critical ratio (CR) values were greater than 3, demonstrating good discriminatory power. Thus, no items were removed at this stage. However, a corrected item-total correlation (CITC) analysis revealed that the CITC value for item XD1 was 0.174, which is below the threshold of 0.3, indicating a weak correlation with other items in the scale. After removing XD1, the Cronbach’s *α* coefficient increased from 0.883 to 0.912, thereby improving the internal consistency of the scale. Additionally, factor analysis using the communality method showed that the extraction communality value for XD1 was only 0.033, which is below the standard threshold of 0.2. Therefore, item XD1 was excluded from the scale.

Following this evaluation, the scale retained 24 items, which were then included in the next stage of analysis.

### 4.2 Exploratory factor analysis

Before conducting the exploratory factor analysis (EFA), the suitability of the data was first assessed. The KMO test yielded a value of 0.926, and Bartlett’s test of sphericity produced a chi-square value with a significance level of less than 0.001. These results confirmed that the data were highly appropriate for EFA. Using principal axis factoring and varimax rotation, we excluded items with factor loadings below 0.4 or cross-loadings below 0.1 to refine the factor structure.

Through multiple rounds of analysis, items RZ1, RZ3, RZ8, QG2, QG4-QG8, XD2-XD3, and XD7 were removed. Ultimately, the EFA extracted two factors with eigenvalues greater than 1, explaining a cumulative variance of 68.765%, which revealed the significant dimensions of the construct being measured. Each factor was named based on the content reflected by its items. Factor 1 consisted of 7 items primarily reflecting students’ cognitive concerns about fitness testing, including worries about failing the test, underperforming, and insufficient regular exercise. These items collectively represent students’ negative cognitive expectations and the associated psychological burden. Therefore, Factor 1 was labeled “Cognitive Anxiety,” emphasizing students’ cognitive appraisals and worries about fitness testing. Factor 2 included 5 items focusing on students’ physiological responses, such as increased heart rate, physical tension, and stomach discomfort. These items highlight the physiological stress reactions students experience before and after the fitness test due to anxiety. Hence, Factor 2 was labeled “Somatic Anxiety,” underscoring the physical responses associated with anxiety. The results of the exploratory factor analysis are presented in Table 2.

**Table 2:** Results of exploratory factor analysis.

Dimension	Item number	Factor loading	Measurement item
Cognitive anxiety	RZ2	0.674	I am worried about failing to meet the passing standards of the fitness test.
	RZ4	0.768	I am afraid of under performing during the fitness test.
	RZ5	0.686	I am concerned that insufficient regular exercise will affect my fitness test results.
	RZ6	0.628	I am worried that inadequate warm-up will impact my test performance.
	RZ7	0.854	I am anxious about not being able to complete the required fitness test items (e.g., 800/1000-meter run, pull-ups, etc.).
	QG1	0.691	I am afraid of accidents occurring during the fitness test (e.g., falling or getting injured).
	QG3	0.597	Thoughts of poor performance during the test distract my attention.
Somatic anxiety	XD4	0.763	Before the fitness test begins, I feel my heart racing.
	XD5	0.721	Standing at the fitness test site, I feel my whole body tense up.
	XD6	0.782	During the fitness test, I feel discomfort in my stomach.
	XD8	0.757	During the fitness test, my palms sweat and feel cold.
	XD9	0.827	During the fitness test, I feel my body stiffen.

### 4.3 Confirmatory factor analysis

#### 4.3.1 Model fit evaluation

To further validate the stability of the revised scale’s internal structure, confirmatory factor analysis (CFA) was conducted in this phase. The sample size in Sample 2 maintained a ratio of approximately 27:1 relative to the number of items, significantly exceeding the minimum required ratio of 4:1, ensuring sufficient statistical power for factor analysis.

**Figure 1:**
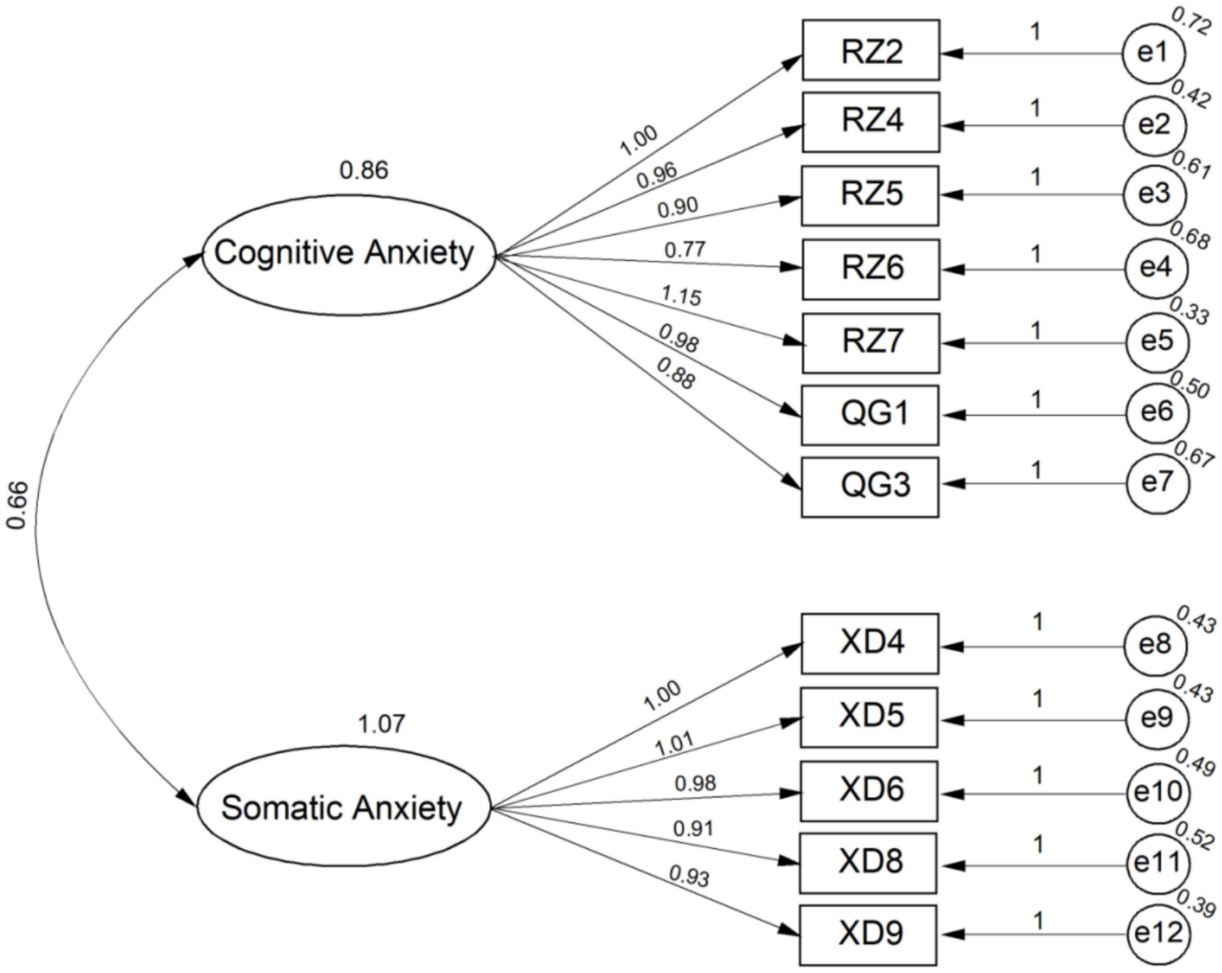
Structure equation model of fitness testing anxiety in university students.

Based on the results of the exploratory factor analysis (EFA), a structural equation model (SEM) was developed. In this model, the 12 items retained from the EFA were included as observed variables, and the two identified factors were defined as latent variables. The purpose of this step was to test the hypothesized relationships between the observed and latent variables and to further verify the construct validity of the scale. Based on established criteria for model fit indices (Browne and Cudeck, 1992; Hu and Bentler, 1999; Kline, 2023), the following thresholds were used: RMSEA <0.06 indicates excellent model fit, and RMSEA between 0.06 and 0.08 indicates acceptable fit. A chi-square to degrees of freedom ratio (*χ*^2^/df) < 3 indicates good fit. Incremental Fit Index (IFI), Comparative Fit Index (CFI), Tucker-Lewis Index (TLI), Goodness of Fit Index (GFI), and Adjusted Goodness of Fit Index (AGFI) > 0.90 indicate good fit, with values closer to 1 indicating better fit. The results (Table 3) showed that all fit indices met the required thresholds, confirming the structural validity of the Fitness Testing Anxiety Scale (FTAS). These findings indicate that the scale effectively reflects the expected factor structure. The model fit results are illustrated in Figure 1.

**Table 3:** Model fit indices for fitness testing anxiety in university students.

Fit index	Fit criteria	Test value	Test result
χ^2^/df	< 5: The model is acceptable	2.290	well
	< 3: The model fits well		
RMSEA	< 0.1: The model is acceptable	0.051	well
	< 0.08: The model fits well		
	< 0.06: The model fits very well		
	< 0.01: The model fits perfectly		
RMR	< 0.1: The model is acceptable	0.045	well
	< 0.05: The model fits well		
IFI	> 0.9: The model fits well	0.980	well
CFI	> 0.9: The model fits well	0.980	well
TLI	> 0.9: The model fits well	0.975	well
GFI	> 0.9: The model fits well	0.955	well
AGFI	> 0.9: The model fits well	0.936	well

#### 4.3.2 Reliability testing

In the reliability analysis, this study evaluated the scale based on the reliability standards proposed by Kline (2013). A high-reliability scale generally requires the Cronbach’s α coefficient of the overall scale to be 0.8 or higher, while the Cronbach’s α coefficient for each subscale should be at least 0.7.The results of this study (see Table 4) showed that the Cronbach’s α coefficient of the overall Fitness Testing Anxiety Scale (FTAS) was as high as 0.928, and the Cronbach’s α coefficients for the individual dimensions ranged from 0.903 to 0.917. This indicates a high level of internal consistency across all dimensions of the questionnaire. Additionally, the split-half reliability values for the overall scale and its dimensions ranged from 0.804 to 0.900, all of which reached statistical significance. This further validated the reliability of the scale in assessing fitness testing anxiety among university students. These results demonstrate that the developed scale exhibits excellent stability and consistency.

**Table 4:** Internal consistency reliability coefficients and split-half reliability for each dimension of the scale.

Dimension	Cronbach’s *α*	Split-half reliability
Cognitive anxiety	0.903	0.900
Somatic anxiety	0.917	0.872
Overall scale	0.928	0.804

#### 4.3.3 Validity testing

Validity testing was conducted from three perspectives: content validity, convergent validity, and discriminant validity. Content validity was evaluated using qualitative methods. The scale was developed based on existing literature and semi-structured interview results. Subsequently, the content sufficiency was assessed, and university students were invited to evaluate the items and complete a small-scale pilot test. These steps ensured a rigorous development process and confirmed the reliability of the scale’s content. Additionally, confirmatory factor analysis (CFA) was conducted to assess validity. As shown in Table 5, all standardized factor loadings exceeded 0.5, the composite reliability (CR) values were greater than 0.7, and the average variance extracted (AVE) values were above 0.5, indicating good convergent validity for the scale. Furthermore, as shown in Table 6, the square roots of the AVE values were all greater than the correlation coefficients in their respective rows and columns, demonstrating that the scale possesses good discriminant validity.

**Table 5:** Convergent validity coefficients for each dimension of the scale.

Dimension	Factor loading	CR	AVE
Cognitive anxiety	0.812	0.904	0.575
Somatic anxiety	0.788	0.909	0.667
Overall scale	0.808	0.950	0.613

**Table 6:** Inter-dimension correlation matrix of the scale (Discriminant validity).

	Cognitive anxiety	Somatic anxiety
Cognitive anxiety	0.758	
Somatic anxiety	0.638	0.816

#### 4.3.4 Criterion-related validity analysis

Criterion-related validity refers to the degree of association between test scores and an external criterion. In this study, the Test Anxiety Inventory (TAI) was employed as the external criterion, as it effectively measures students’ anxiety levels in evaluative testing environments (Speilberger, 1980), which aligns conceptually with the construct of physical fitness test anxiety. Pearson correlation analysis was conducted to examine the relationships between the Physical Fitness Test Anxiety Scale for University Students and the overall TAI score as well as its subdimensions. The correlation analysis revealed that the total score of the Physical Fitness Test Anxiety Scale for University Students was significantly positively correlated with the total score of the TAI (*r* = 0.487, *p* < 0.001). Additionally, significant positive correlations were observed with the cognitive dimension of the TAI (*r* = 0.435, *p* < 0.001) and the emotional response dimension (*r* = 0.402, *p* < 0.001). Furthermore, the scale demonstrated significant correlations with anxiety-related factors from the Competitive State Anxiety Inventory-2 (CSAI-2) (*r* = 0.364, *p* < 0.01).These findings indicate that the Physical Fitness Test Anxiety Scale for University Students exhibits significant positive correlations with theoretically relevant external criteria, thereby supporting the criterion-related validity of the scale.

## 5 Discussion

### 5.1 Research conclusions and contributions

This study followed standardized scale development procedures to construct and validate the Physical Fitness Test Anxiety Scale (FTAS) tailored for Chinese university students, providing a reliable tool for assessing anxiety levels related to physical fitness tests. The key conclusions are as follows:

First, through literature analysis and semi-structured interviews, an initial item pool was developed, clarifying the core conceptualization of physical fitness test anxiety and laying a theoretical foundation for scale development. This study innovatively integrated the context of China’s National Student Physical Fitness Standard (NSPFS) system, proposing a dual-dimensional framework of cognitive and somatic anxiety. This framework provides a theoretical basis for understanding the characteristics of university students’ anxiety in standardized testing contexts.

Second, the initial items were refined through expert reviews and pilot testing. Item analysis and exploratory factor analysis (EFA) were conducted to eliminate unsuitable items, resulting in a final scale comprising two dimensions—cognitive anxiety and somatic anxiety—with a total of 12 items. This dual-dimensional structure adheres to psychometric standards and comprehensively reflects university students’ experiences of physical fitness test anxiety, demonstrating high situational applicability and cultural relevance.

Third, confirmatory factor analysis (CFA) was employed to examine the scale’s structural validity, and its reliability and validity were scientifically verified using multiple indicators, including internal consistency coefficients, split-half reliability, convergent validity, discriminant validity, and criterion-related validity. The results indicate that the FTAS exhibits good model fit and can stably and effectively measure university students’ anxiety levels during physical fitness tests.

In summary, the FTAS developed in this study holds significant theoretical and practical implications. Theoretically, it innovatively constructs a dual-dimensional conceptual framework for physical fitness test anxiety. Methodologically, it provides a concise and psychometrically sound measurement tool. Practically, it offers a scientific basis for universities to assess and intervene in physical fitness test anxiety. This scale addresses the gap in specialized assessment tools for university students’ physical fitness test anxiety in China, providing critical scientific support for future research on the mechanisms of physical fitness test anxiety, university mental health interventions, and the optimization of physical fitness testing evaluation systems.

### 5.2 Research limitations and future research prospects

Although this study provides a reliable tool for measuring university students’ physical fitness test anxiety, several limitations remain. Future research could address these limitations and further expand and deepen the understanding of this construct through the following strategies:

First, the sample in this study primarily consisted of university students from a specific region. While the sample size (item-to-response ratio of 27:1) met the requirements for statistical analysis, it did not sufficiently encompass students from diverse geographical regions, physical fitness levels, and cultural backgrounds (e.g., differences between sports majors and non-sports majors). This may limit the generalizability of the scale. Future studies should expand the sampling scope, conduct multi-center collaborations, and include students from urban and rural areas, as well as universities in different climate zones, to enhance sample diversity. Additionally, independent sample groups, such as students who have repeatedly failed physical fitness tests or those with athletic specialties, could be established to validate the scale’s cross-group measurement equivalence through multi-group analysis.

Second, the cross-sectional design of this study did not track the dynamic changes in students’ anxiety before, during, and after physical fitness tests. Moreover, although the scale demonstrated good reliability and validity, reliance on self-reported data may introduce social desirability bias. Future research should adopt longitudinal designs, covering different time points within the testing cycle (e.g., one month before the test, one week before the test, the day of the test, and 24 h after the test) to capture the dynamic patterns of anxiety. Additionally, items such as “The closer the physical fitness test date, the more nervous I feel” could be developed to reveal fluctuations in anxiety throughout the testing cycle. Objective measures, such as physiological indicators (e.g., heart rate, blood pressure, skin conductance), or multi-source data (e.g., teacher evaluations, peer evaluations) could also be incorporated to enhance measurement accuracy.

Third, this study primarily focused on the cognitive and somatic dimensions of physical fitness test anxiety. While these dimensions effectively explain the main features of physical fitness test anxiety, the complex phenomenon of anxiety may also include other dimensions, such as self-confidence, social evaluation, etc. (Martens et al., 1990; Smith et al., 1990). Previous studies have shown that individuals with high self-confidence can better maintain psychological stability and adopt positive coping strategies in anxiety situations, while individuals with low self-confidence are more vulnerable to the negative impact of anxiety (Watson et al., 2021). In addition, students’ worry about the negative evaluation of teachers and peers may increase the level of test anxiety (Li et al., 2023; Tsarpalis-Fragkoulidis et al., 2024). Future studies could incorporate these dimensions into the model, exploring a multi-dimensional construct of physical fitness test anxiety. By introducing latent variables such as self-confidence and social evaluation, a more comprehensive theoretical framework could be established to better understand the mechanisms underlying physical fitness test anxiety and its multifaceted impact on students’ psychology and behavior.

Fourth, while this study validated the basic psychometric properties of the scale, a standardized scoring system has yet to be established. Future research could develop normative data through large-scale testing, scientifically categorizing physical fitness test anxiety into different levels (e.g., low, moderate, high) and establishing scoring criteria and cut-off values for each dimension. Additionally, considering potential differences in physical fitness test anxiety across demographic variables such as gender and academic year, future studies could explore the moderating effects of these variables and establish group-specific scoring references. This would enhance the practical application value of the scale in educational settings, providing more precise assessment tools for university physical education and psychological counseling.

## Data Availability

The original contributions presented in the study are included in the article/supplementary material, further inquiries can be directed to the corresponding author/s.
